# Determining Microeukaryotic Plankton Community around Xiamen Island, Southeast China, Using Illumina MiSeq and PCR-DGGE Techniques

**DOI:** 10.1371/journal.pone.0127721

**Published:** 2015-05-28

**Authors:** Lingyu Yu, Wenjing Zhang, Lemian Liu, Jun Yang

**Affiliations:** 1 Laboratory of Marine Biodiversity and Global Change (MBiGC), College of Ocean and Earth Sciences, Xiamen University, Xiamen, China; 2 Aquatic EcoHealth Group, Key Laboratory of Urban Environment and Health, Institute of Urban Environment, Chinese Academy of Sciences, Xiamen, China; National Cheng-Kung University, TAIWAN

## Abstract

Microeukaryotic plankton are important components of aquatic environments and play key roles in marine microbial food webs; however, little is known about their genetic diversity in subtropical offshore areas. Here we examined the community composition and genetic diversity of the microeukaryotic plankton in Xiamen offshore water by PCR-DGGE (polymerase chain reaction-denaturing gradient gel electrophoresis), clone-based sequencing and Illumina based sequencing. The Illumina MiSeq sequencing revealed a much (approximately two orders of magnitude) higher species richness of the microeukaryotic community than DGGE, but there were no significant difference in species richness and diversity among the northern, eastern, southern or western stations based on both methods. In this study, Copepoda, Ciliophora, Chlorophyta, Dinophyceae, Cryptophyta and Bacillariophyta (diatoms) were the dominant groups even though diatoms were not detected by DGGE. Our Illumina based results indicated that two northern communities (sites N2 and N3) were significantly different from others in having more protozoa and fewer diatoms. Redundancy analysis (RDA) showed that both temperature and salinity were the significant environmental factors influencing dominant species communities, whereas the full microeukaryotic community appeared to be affected by a complex of environmental factors. Our results suggested that extensive sampling combined with more deep sequencing are needed to obtain the complete diversity of the microeukaryotic community, and different diversity patterns for both abundant and rare taxa may be important in evaluating the marine ecosystem health.

## Introduction

Microeukaryotic planktonic microbes are ubiquitous and diverse in marine ecosystems. These microorganisms, including protozoa, algae, fungi and small metazoa are important components of both traditional marine food webs and the microbial food webs [1 **–** 2]. Small variations in the structure and composition of these communities will usually result in significant changes at all trophic levels [3]. Normally, most microeukaryotic plankton assemblages, such as phytoplankton, are susceptible to environmental deterioration and pollution. Therefore, these microorganisms make ideal biomonitors to evaluate the ecosystem status of marine waters [3–4]. As predators, producers, decomposer and parasites, the microeukaryotic microorganisms play key roles in the ecological functioning and process of biological marine ecosystems [5 **–** 6]. It is therefore important to study the diversity in microeukaryotic communities to characterize both biogeochemical processes and marine ecosystem health.

Microscopic identification and quantification has traditionally been used in the study of microeukaryotic plankton communities. However, there are some limitations [7], because microorganisms are sometimes too small and lacking distinctive and rigorous taxonomic features for reliable microscopic identification [8]. Therefore, more effective techniques should be introduced into the study of microbial diversity and ecology. Over the past two decades, molecular methods have been increasingly applied to investigations of microeukaryotic communities from natural ecosystems, and sequencing ribosomal RNA (rRNA) gene has become a standard method for assessing microeukaryotic diversity [5]. For example, PCR**-**DGGE (polymerase chain reaction-denaturing gradient gel electrophoresis) has been routinely applied in microbial ecology studies and this method has been confirmed to be an efficient approach to the detection of microeukaryotic community dynamics in marine ecosystems [3–4]. Recently, next**-**generation sequencing (NGS) has become the most promising approach in the study of the ecology and diversity of microeukaryotic planktonic communities, targeting hypervariable regions such as V2**-**V3, V4 or V9 of the small subunit (SSU) rRNA gene [5, 9]. In this study, both PCR**-**DGGE and the MiSeq platform sequencing (NGS from Illumina) were performed to characterize the microeukaryotic plankton communities in Xiamen offshore waters.

Xiamen is an important marine port located in the southeast of China. The southeast of Xiamen Island faces to Taiwan Strait also referred to as the ‘sea corridor’ of the East China Sea, and the southwest of it is the Jiulong River Estuary [10]. Moreover, the coastal sea around Xiamen serves as an important sea area for transportation, tourism and fishery in Fujian province. However, only few studies have been conducted on the diversity and dynamics of microeukaryotic plankton assemblages in this area [11], and so the composition and structure of microeukaryotic communities are not fully understood. The understanding of microeukaryotic communities, and their relationship with environmental factors, is necessary to better manage and protect the local coastal ecosystems.

Our study aimed at (1) using both Illumina high throughput sequencing and PCR**-**DGGE techniques to explore the diversity and composition of microeukaryotic communities in subtropical Xiamen offshore area; and (2) revealing the environmental factors which influence the microeukaryotic community distribution in Xiamen coastal waters.

## Materials and Methods

### Sample collection and DNA extraction

**Fig 1:**
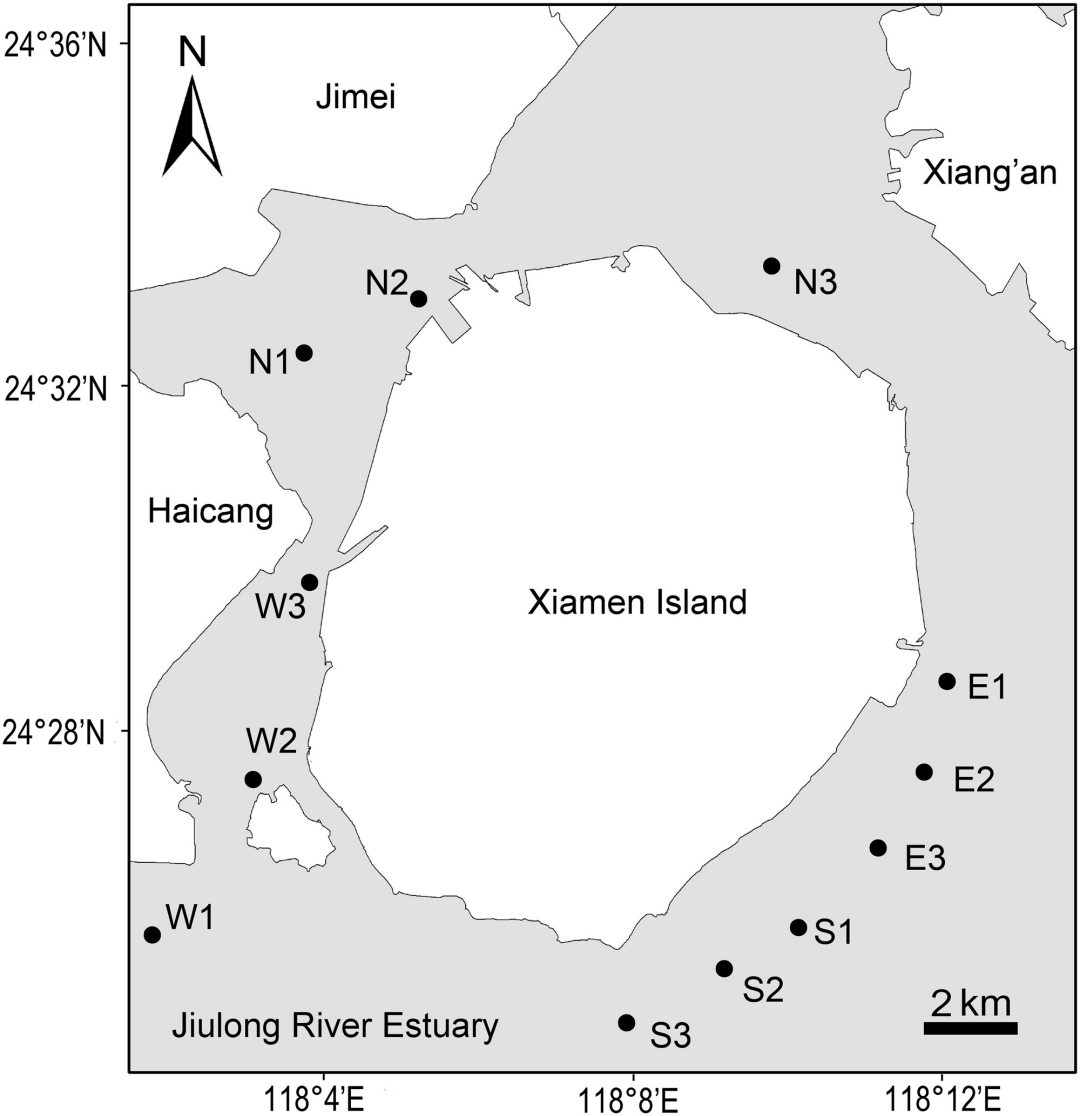
Map of Xiamen offshore sea area showing the locations of twelve sampling sites.

Xiamen is an island city surrounded by coastal sea. A field cruise was carried out around Xiamen offshore area (118°01'–118°14' E, 24°25'–24°34' N) in July 2013. The climate of Xiamen is subtropical maritime monsoon, and the mean annual temperature and precipitation are 21°C and 1200 mm, respectively. Surface seawater (< 1 m depth) samples were collected from 12 Xiamen coastal offshore sites (Fig 1), and three samples were taken from north (N), east (E), south (S) and west (W) stations of Xiamen Island offshore area, respectively. In this field sampling, no particular permissions were required because this scientific research did no harm to sea environment, and no protected or endangered groups were involved in this investigation. For the DGGE and MiSeq sequencing analysis of microeukaryotic plankton communities, 800 ml of surface seawater was pre**-**filtered by a 200 μm sieve to remove debris, meso**-,** macro**-**plankton, and next filtered through 0.22 μm pore polycarbonate membrane (Millipore, Billerica, MA, USA). After that, the membranes were frozen at **-**80°C for further process.

Total nucleic acids were extracted by E.Z.N.A. DNA Kit (Omega Bio-Tek Inc., Norcross, GA, USA) following standard instructions. All DNA extracts were eluted with 50 μL TE buffer and stored at -20°C until further analyses. DNA quality and concentration were measured by Nano-Drop 1000 spectrophotometer (Thermo Fisher Scientific Inc., Wilmington, DE, USA).

### Environmental factors

Seawater temperature, salinity, pH, DO (dissolved oxygen), Chl**-**a (chlorophyll a) and turbidity were all measured *in situ* using a Hydrolab DS5 multiparameter water quality meter (Hach Company, Loveland, CO, USA). In the laboratory, TN (total nitrogen) was analyzed with a TOC/TN**-**VCPH analyzer (Shimadzu, Tokyo, Japan) and TP (total phosphorus) was determined using spectrophotometry according to standard methods [2].

### PCR-DGGE and clone sequencing

The 18S rRNA gene was amplified from the microeukaryotic communities using the primers Euk516r with a ‘GC’ rich sequence and Euk1A [12]. 50 μL PCR mixtures contained 5 μL of 10× reaction buffer, 200 μM dNTP, about 40 ng target DNA, 0.3 μM of forward-reverse primer, 2.5 U Ex-Taq polymerase (Takara, Otsu, Shiga, Japan) and 1.5 mM MgCl_2_. ‘Touch-down’ PCR process was as follows: an initial 10 min denaturing step at 94°C, then ten times touchdown steps including denaturation at 94°C for 30 seconds, then 30 seconds annealing start at 67°C (by temperature reducing 1°C once), extension step at 72°C for 1 min. Furthermore 25 additional cycles including 94°C for 30 seconds, 57°C for 30 seconds and 72°C for 1 min were carried out. Final extension step was carried out at 72°C for 10 min. Next, the PCR products were inspected in 1.0% agarose gel, then dyed using SYBR Green I and viewed under ultraviolet transilluminator according to Liu et al [3].

We used the Dcode Universal Mutation Detection System (BioRad, Hercules, CA, USA) for the sequence-specific separation of the PCR products. Twenty**-**five microliters of each PCR product was added into 6% (w/v) polyacrylamide gel with 25%**-**45% denaturing gradient. The running buffer was TAE (40 mM Tris, 40 mM acetic acid and 1 mM EDTA, pH 7.4). We performed electrophoresis at 60°C and 100 V for 16 h. Then the gel was stained with SYBR Green I for about 20 min, carefully washed by distilled water, and finally photographed using Ettan DIGE Imager (GE Healthcare, Piscataway, NJ, USA). Prominent DGGE bands were carefully cut from the gel and stored into aseptic tubes, adding thirty microliters sterilized distilled water and deposited at 4°C overnight. Six microliters eluted DNA was subjected to a new amplification with the same primers Euk1A and Euk516r (without GC clamp). The *Escherichia coli* DH5α competent cells (Takara, Otsu, Shiga, Japan) and pGEM**-**T Easy Vector (Promega, Madison, WI, USA) were employed in clone process. Finally, in order to obtain the 18S rRNA gene information we picked three colonies per band for sequencing. The inserted plasmids sequencing process was performed by an ABI3730 DNA Analyzer (Applied Biosystems, Foster City, CA, USA).

### Illumina MiSeq sequencing

Total DNA from twelve sites was sent to Novogene Co., Ltd. Beijing for Illumina MiSeq sequencing using a paired**-**end 150 bp sequence read run with the Miseq Reagent Kit v3. A set of primers were applied to amplify hypervariable V9 region in eukaryotic 18S rDNA. In this study, the forward and reverse primers were 1380F and 1510R [13]. Twelve DNA samples were independently amplified in triplicated 25 μL reactions including 5 min denaturation at 94°C, then 25 cycles of 30 seconds at 94°C, 30 seconds at 50°C and 30 seconds at 72°C. The final amplification was 7 min extension at 72°C. In total 1 × PCR buffer, 0.625 U Ex**-**Taq polymerase, 2.5 mM dNTPs, 20 ng target DNA and 10 μM each primer were included in 25 μL reaction system. The FLASH [14] was used to merge reads pairs from DNA fragments. Quality**-**checked sequences were analyzed by QIIME v1.7.0 (Quantitative Insights Into Microbial Ecology) [15], where data procedure were as follows: a) maximum of continuous low**-**quality base is three; b) minimum of continuous high**-**quality base is 75% of total read length; c) no ambiguous (N) character exists in sequences; d) last quality score is three. Standardized sequence number of twelve samples was selected randomly [16]. After that, we used UPARSE [17] to pick OTUs (operational taxonomic units) by constructing OTU table. All reads were assigned into OTUs at 97% similarity threshold by uclust v1.2.22q [18]. One representative sequence of each OTU was selected and matched with SILVA [19] sequences by RDP [20] classifier to identify phylogenetic affiliation [21 **–** 22]. Chimera and singleton sequences were discarded prior to further analysis [18].

### Data analysis

DGGE profiles were analyzed by Quantity One (BioRad, Hercules, CA, USA). The software can automatically detect the bands, besides the results can be manually checked. DGGE bands profile and Miseq sequencing standardized OTUs were transformed into binary code, where ‘1’ and ‘0’ indicated ‘presence’ and ‘absence’, respectively. Two Bray**-**Curtis similarity matrices based on DGGE and Illumina data were constructed, respectively. Then the non**-**metric multidimensional scaling analysis (MDS) was employed for detecting patterns in microeukaryotic communities among north, east, south and west stations. Significant difference (*P* < 0.01) between groups was assessed by analysis of similarities (ANOSIM). The global R statistic ranges from 0 to 1 represents separation degree between site groups, and no separation is indicated by R = 0, while R = 1 indicates complete separation [23].

All physicochemical parameters except pH were square**-**root transformed for improving homoscedasticity and normality [24]. After that, preliminary DCA (detrended correspondence analysis) on biological data was applied to decide whether linear or unimodal ordination methods should be used. The DCA result based on DGGE data showed the longest gradient length was less than 3.0, while DCA on Miseq sequencing OTU data revealed that longest gradient length was between 3.0 and 4.0, thereby implying most taxa displayed linear responses to environmental factors. Therefore, we chose redundancy analysis (RDA) to examine the relationship between microeukaryotic community and environmental factors.

Shannon**-**Wiener index (*H’*) was calculated based on microeukaryotic DGGE profiles and Miseq sequencing OTUs. The *H’* was calculated using the following equation: *H’* = **-**Σ*P*_*i*_ln*P*_*i*_. The term *P*_*i*_ was determined by the equation: *P*_*i*_ = *n*_*i*_/*N*, where *n*_*i*_ is the number of *i*th band or OTU in a sample and *N* is the sum of the band or sequence numbers. Both Scheffe’s *F* multiple**-**comparison test and ANOVA (analysis of variance) were employed to detect differences among twelve stations. Statistical analyses were run in STATISTICA 6.0, PRIMER 5.0, ORIGIN 8.0, CANOCO 4.5 and the R software packages.

The Z score was calculated for the relative abundance of microbial eukaryotes from Miseq sequencing heatmap. The Z score was determined using the equation: $\text{Z}=\;({\text{x}}_{\text{i}}-\bar{\text{x}})/\text{SD}$. Where x_i_ is relative abundance of a certain microeukaryote, and $\bar{\text{x}}$ is average relative abundance in all sampling sites.

### Accession number

The 18S rRNA gene sequences were submitted to the GenBank under the accession numbers KJ638859–KJ638888, KR019689–KR019736. All MiSeq Platform sequencing data was deposited in the SRA (sequence read archive) of public NCBI database under the accession number SRX651777 (http://www.ncbi.nlm.nih.gov/).

## Results

### Environmental characteristics

Eight physiochemical variables describing the surface water are briefly summarized in Table 1. The temperature in the north sites (N1–N3) was significant higher than that of south, east and west sites (t**-**test, *P* = 0.03). Salinity in the southeast sites except S3 was influenced by open sea, and it was higher than that of north and west sites (t**-**test, *P* = 0.01). Ranges for pH, DO and Chl**-**a were 8.99**–**9.80, 5.42**–**13.06 mg l^-1^ and 1.88**–**26.85 μg l^-1^, respectively. The concentrations of TN were higher in W1, W2 and S3 sites, which are closed to the Jiulong River Estuary. The TP concentration ranged from 0.013 to 0.025 mg l^-1^.

**Table 1:** Environmental parameters in the twelve sampling sites of Xiamen offshore area.

	N1	N2	N3	E1	E2	E3	S1	S2	S3	W1	W2	W3
Temperature (°C)	30.22	31.51	31.67	28.47	28.89	29.18	28.31	28.13	29.33	29.38	28.94	29.22
Salinity (psu)	25.9	26.5	27.4	30.9	30.8	30.5	30.7	30.1	21.3	12.6	23.4	26.6
pH	9.26	9.40	9.80	9.16	9.28	9.31	9.42	9.54	9.29	8.99	9.30	9.42
DO (mg l^-1^)	6.30	7.56	13.06	5.65	6.02	6.21	6.90	9.07	6.27	5.42	6.28	7.78
Chl-a (μg l^-1^)	3.40	5.03	26.85	2.64	2.89	3.18	9.19	8.08	1.88	4.70	5.05	10.41
Turbidity (NTU)	9.9	11.1	11.6	21.9	16.3	13.5	12.9	8.2	9.3	20.8	11.0	8.3
TP (mg l^-1^)	0.021	0.019	0.025	0.016	0.016	0.014	0.018	0.013	0.019	0.021	0.019	0.019
TN (mg l^-1^)	0.710	0.370	0.570	0.400	0.410	0.330	0.220	0.350	0.830	1.340	0.780	0.490

### Microeukaryotic communities revealed by PCR-DGGE

DGGE profiles of microeukaryotic community displayed high difference in bands position among the twelve lanes (S1 Fig). In total 73 distinct bands were revealed using DGGE fingerprinting analysis. There were four bands (5.5%) that were prevalent at all sites and five unique bands (6.8%) which were only present at one site. The number of bands ranged from 26 to 39 in 12 sites, with a mean of 30. Sites E1 and W1 had the greatest number of bands (39), whereas sites W2, N1 and N2 showed the lowest band number (26). The Shannon**-**Wiener index ranged from 1.34 to 1.59 (mean 1.48).

**Fig 2:**
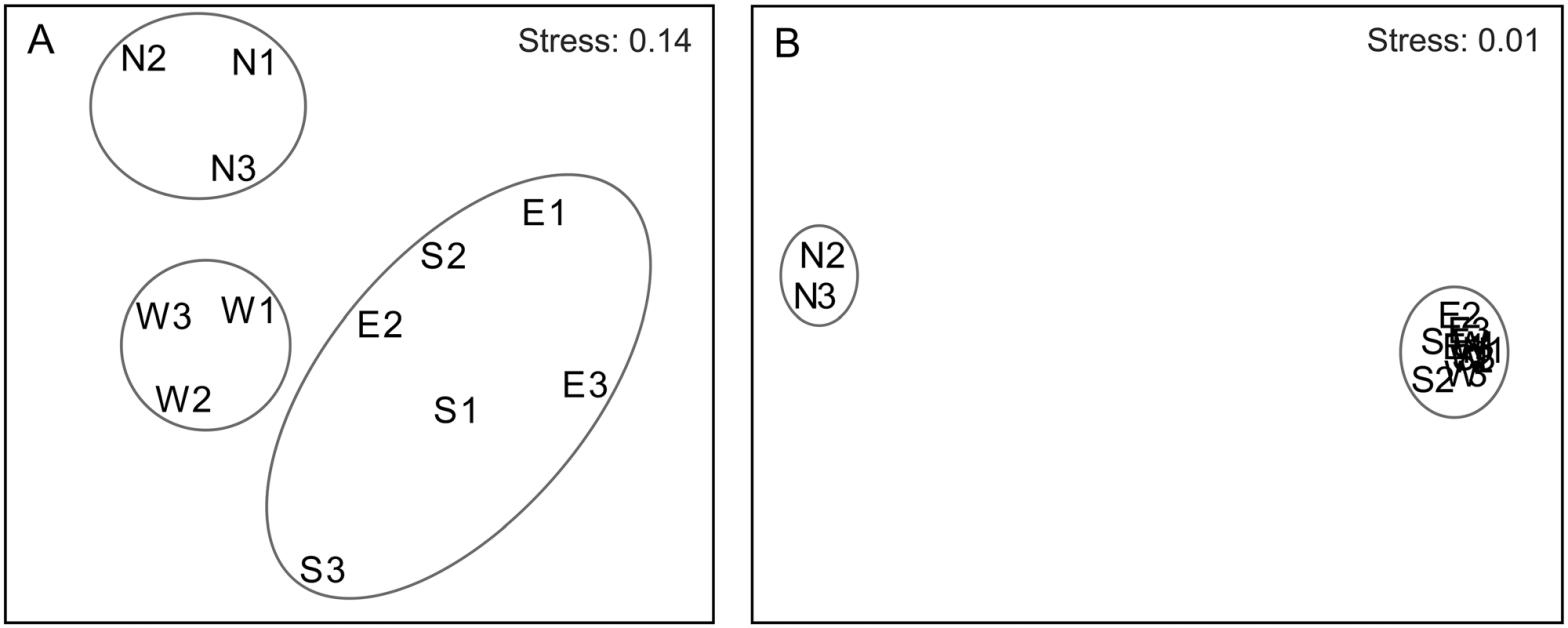
MDS ordination of microeukaryotic communities based on DGGE profiles (A) and Illumina Miseq data (B).

**Fig 3:**
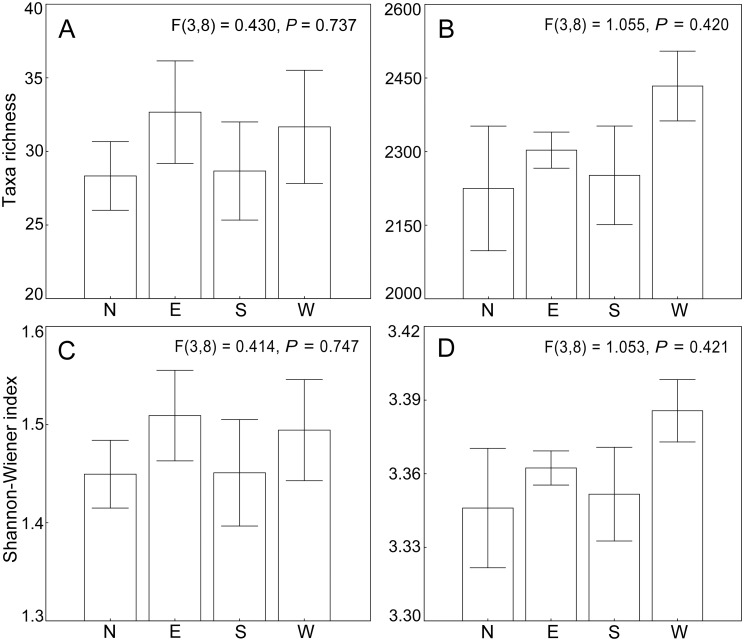
DGGE and Illumina Miseq based diversity of microeukaryotic communities from Xiamen offshore sea. DGGE band number (A) and Illumina Miseq based OTU number (B), Shannon–Wiener index based on DGGE profiles (C) and Miseq sequencing data (D). (N, northern sites; E, eastern sites; S, southern sites; W, western sites). Values and error bars indicate mean and standard error.

Clearly, three groups were divided by MDS for microeukaryotic communities (Fig 2A). Group I was composed of north sites 1**–**3, while Group II was clustered with west sites 1**–**3. Group III consisted of east sites 1**–**3 and south sites 1**–**3. This was also supported by the ANOSIM analysis, revealing a strong spatial separation of the microeukaryotic community among the three groups (R = 0.619, *P* = 0.001). The average band numbers were 28, 33, 29 and 32 in north, east, south and west sites, respectively. The average Shannon**-**Wiener indices were 1.45, 1.51, 1.45 and 1.49 in north, east, south and west sites, respectively. However, there was no significant difference in the band numbers (*P* > 0.05) and Shannon**-**Wiener index (*P* > 0.05) among communities from north, east, south and west sampling sites (Fig 3A and 3C).

**Fig 4:**
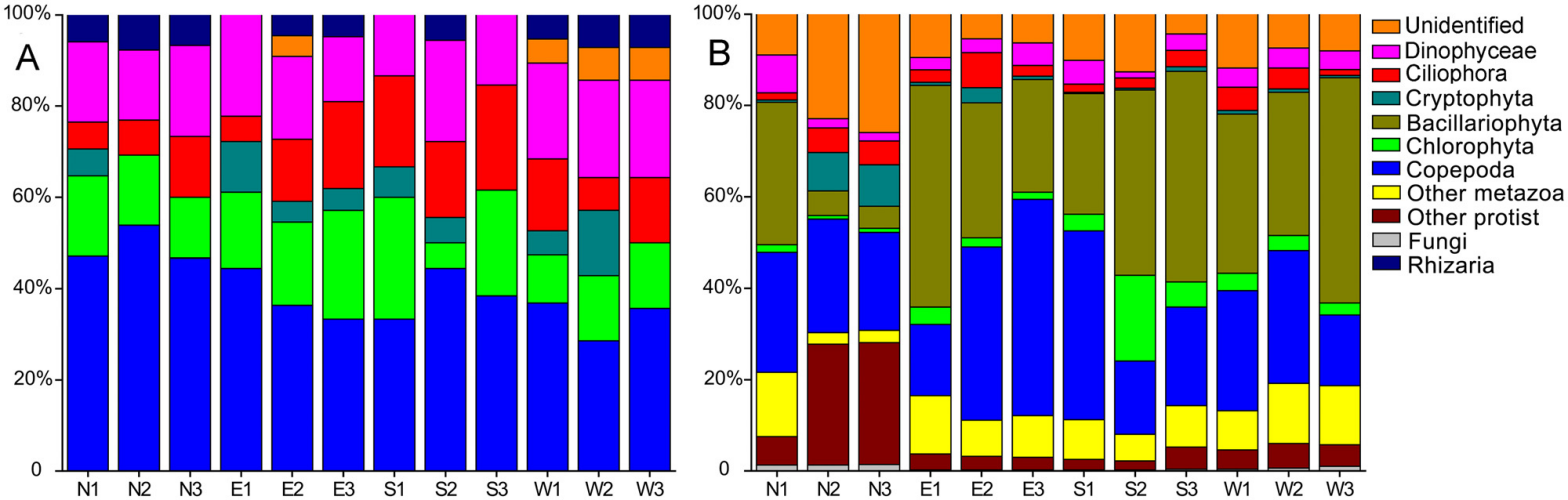
Percentage of sequences in all OTUs classified by major taxonomic groups of twelve sampling sites**,** as revealed by (A) DGGE and clone based sequencing and (B) Illumina Miseq sequencing.

In total, 26 dominant DGGE bands were successfully re**-**amplified and cloned (S1 Fig). After sequencing, we achieved 78 sequences and 21 OTUs at 97% similarity level (S1 Table). Seven major microeukaryotic groups (Copepoda, Ciliophora, Chlorophyta, Dinophyceae, Cryptophyta, Rhizaria and unknown taxa) were identified, and Copepoda (39.9%) was the most dominant taxon (Fig 4A). Fourteen microeukaryotic OTUs belonged to the uncultured species from marine ecosystems. Further, we found four bands (B28, B31, B32 and B35 harbored two different taxa, respectively. However, five OTUs (OTU1, OTU2, OTU4, OTU6, and OTU15) were distributed in more than two different bands on the DGGE gel (S1 Table).

### Microeukaryotic communities revealed by Illumina MiSeq sequencing

From the MiSeq run, we got 923,718 raw sequences reads and 886,637 high**-**quality reads from 12 samples. After applying quality control procedures, the number of reads ranged from 47,891 to 104,954 per sample. After deleting the chimera sequences and singletons (OTUs containing a single read across all samples), we randomly selected 47,493 reads from each sample to normalize the sampling effort, resulting in a total 569,916 sequences for further analyses. After standardization, we gained 8,983 different OTUs by clustering with UPARSE. The top five most abundant OTUs in each sampling site of Xiamen offshore waters were summarized, and in total we listed 60 OTUs, 51.67% of which showed more than 97% similarity with certain species in the GenBank database (S2 Table). The phylum, OTU, sequence number of metazoa, protist, fungi and unclassified species in twelve sampling sites are showed in S3 Table. The OTU number of protist, ranged from 1,143 to 1,665 (mean 1,377 OTUs), was far more than that of metazoa (235–396 OTUs), fungi (13–74 OTUs) and unclassified species (433–673 OTUs). In addition, protist phylum number ranged from 15 to 22 and had a mean of 18 phyla per sample. We performed rarefaction analysis on 12 samples and found that none of our samples showed a full saturation in rarefaction curve. Interestingly, estimates of taxa richness from our rarefaction analysis were similar in all samples ranging from 2,066 to 2,554. However, we conducted further rarefaction analysis with the total pooled data set and produced an about four**-**fold taxa richness (8,983) of a single site (S2 Fig).

In general, taxonomic composition sites of N2 and N3 showed a highly different trend from other sites (Fig 2B). The average Miseq sequencing OTU numbers in north, east, south and west sites were 2,225, 2,303, 2,252 and 2,434, respectively. The average Shannon**-**Wiener indices in north, east, south and west sites were 3.35, 3.36, 3.35 and 3.39, respectively. However, there was still no significant difference in the OTU number (*P* > 0.05) and Shannon**-**Wiener indices (*P* > 0.05) among north, east, south and west sampling sites (Fig 3B and 3D). We obtained a distinct pattern of taxonomic composition, which was made up by different percentages of major taxonomic groups (Fig 4B). The ubiquity of Copepoda was obvious, because it accounted for a larger percentage (mean 27.14%) among the twelve sampling sites. The diatoms (Bacillariophyta) were also prevalent, although they showed a lower proportion at sites N2 and N3. The microeukaryotic communities at sites N2 and N3 were significantly different from those at 10 other sites (R = 1.000, *P* = 0.005) because there were a large proportion of unclassified taxa and other protists at both sites. It was interesting that some ciliates such as *Rimostrombidium*, *Halteria*, *Tintinnidium*, *Cryptocaryon* and *Pseudotontonia* were more abundant in both sites N2 and N3 compared to other sites (S3 Fig).

### Relationship between environmental factors and microeukaryotic communities

**Fig 5:**
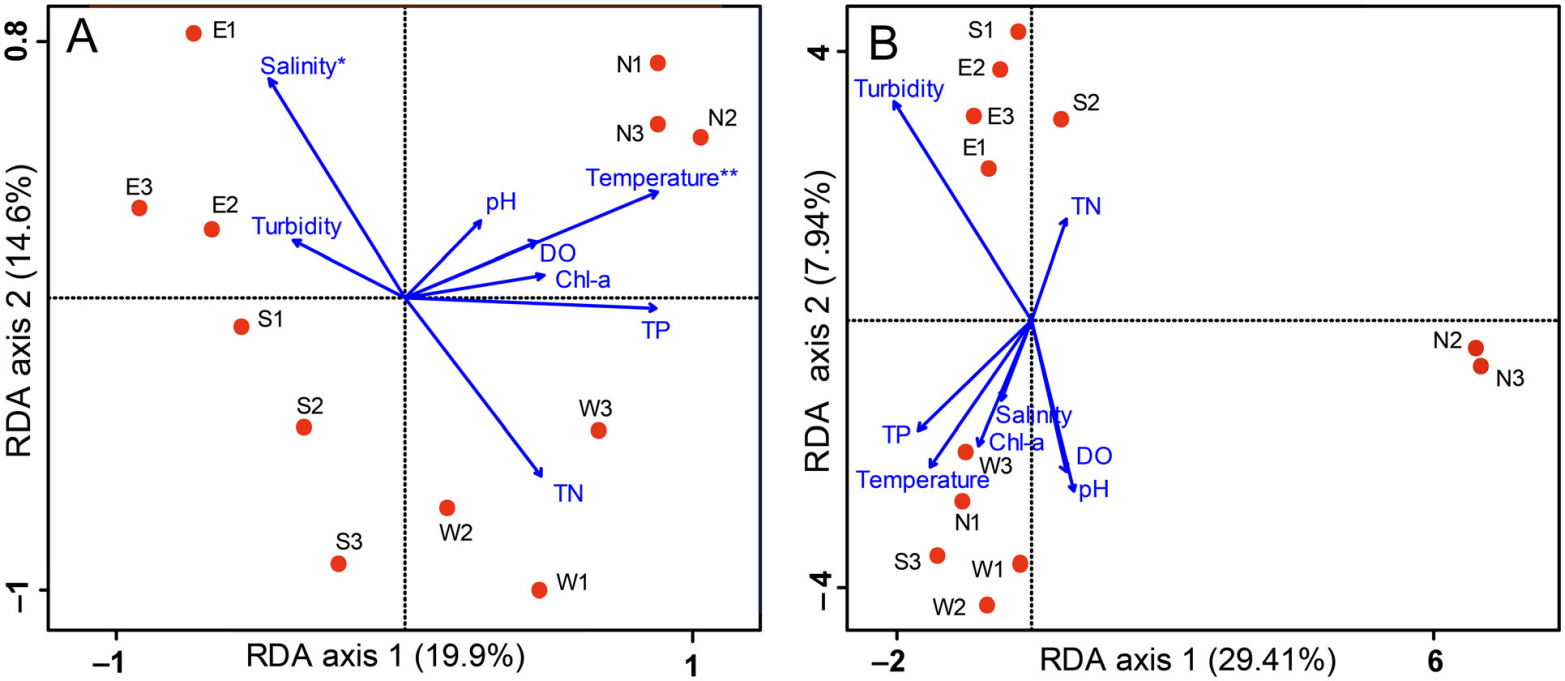
RDA ordination showing the microeukaryotic community composition in relation to the environmental variables. The community composition was based on DGGE (A) and Miseq sequencing (B), respectively. Sampling sites are displayed by circles, environmental variables are shown by arrows. Statistically significant environmental variables are marked with an asterisk (*P* < 0.1) and two asterisks (*P* < 0.05) according to Monte Carlo permutation test.

Our RDA analysis based on DGGE and Miseq Sequencing data showed an obvious difference. It was revealed that the DGGE fingerprints were significantly related to the temperature (*P* < 0.05). The axes 1 and 2 explained 19.9% and 14.6% of the community data variability, respectively (Fig 5A). Temperature and TP were positively correlated with axis 1, with correlation coefficients 0.799 and 0.795, respectively. Salinity was found to be related (*P* < 0.1) to composition of the microeukaryotic community with the correlation coefficient **-**0.692.

Fig 5B displayed the relationship between Illumina**-**based microeukaryotic communities and environmental variables. The axes 1 and 2 explained 29.41% and 7.94% of the community data variability, respectively. Surprisingly, no significant variable was detected in this RDA analysis.

## Discussion

### Influence of environmental factors

Our results indicated that the DGGE**-**based microeukaryotic communities relate to environmental changes in seawater. In fact, in the transitional area of Jiulong River and Xiamen Sea water chemistry was significantly influenced by the Jiulong River watershed which is characterized by severe agricultural pollutions and industrial activities [10]. Both chemical fertilizer usage and livestock production make the Jiulong River Watershed a huge source of nitrogen and phosphorus, thereby exporting a large amount of nutrient pollutants into Xiamen offshore waters [10, 25]. On the whole, the community composition of microeukaryotes was similar in the eastern and southern sites, but distinct from that of western and northern sites (Fig 2A). This difference was closely related to agricultural pollution and high nitrogen freshwater from the Jiulong River, because the transitional area, especially sampling sites S3, W1 and W2 showed a higher concentration of total nitrogen (mean 0.983 mg l^-1^) and lower salinity (mean 19.1 psu) (Table 1). It is acknowledged that nutrient concentrations can affect microeukaryotic community because different microeukaryotic organisms are well adapted to their preferable nutritional conditions [26]. Nutrients can influence the photosynthesis of phytoplankton directly, while heterotrophic microeukaryotes can be impacted by nutrients through the effect on phytoplankton growth [27]. In the Jiulong River, both total nitrogen and NH_4_ **-**N were significant environmental parameters influencing microeukaryotic communities distribution in spatial level [3].

The microeukaryotic communities revealed by Miseq sequencing were also affected by the transitional sea area. For example, western sites harboured a higher taxon richness and diversity than other sites—although not significant so (Fig 3B and 3D). One possible reason is that western sites were located in a transition zone between freshwater and marine environments and such complex and dynamic environments can provide more suitable conditions for diverse microeukaryotic plankton [3]. In fact, the western sites were influenced by the Jiulong River and Yundang Lake, which is the largest brackish lake in Xiamen Island. The frequent water exchange with the Jiulong River and Yundang Lake could contribute a certain amount of freshwater and brackish species to west sites.

Interestingly, our MDS result displayed that three north microeukaryotic plankton communities were very different from the communities in other sites. The RDA showed that northern sites were mainly related to temperature, which appeared to be significantly related to microeukaryotic plankton communities revealed by DGGE (*P* < 0.05). In fact, temperature in the northern sites ranged from 30.22°C to 31.67°C showing a higher average value than that of other sites. Further, the RDA revealed by DGGE demonstrated that the dominant species in Xiamen offshore sea area have a connection with salinity. Salinity is an important environmental variable to the survival, growth and development of marine microeukaryotic plankton [28]. The impacts of salinity on microeukaryotic plankton groups are usually related with cell response to metabolic or osmoregulation changes [29]. In our study, salinity was found to be related to composition of the microeukaryotic community (*P* < 0.1), its value was comparatively higher in sites E1, E2, E3, S1 and S2 than those in other sites, because these five sites were more exposed to ocean current from the Taiwan Strait. In contrast, S3, W1 and W2 sites were largely affected by the diluted freshwater from the Jiulong River, thereby showing the lowest salinity value among 12 sampling sites.

The Illumina-based RDA result showed a clear difference from that revealed by DGGE. However, no significant variable was detected to be related with microeukaryotic community composition revealed by Miseq sequencing, indicating that the entire community might correlate with a complex of environmental factors or other mechanisms. It is also possible that microeukaryotic phylotypes might correlate with other environmental variables including water flow and bacterioplankton [30], and grazing pressure which were not investigated in our study. Miseq sequencing result also contained a number of rare species, opposite to abundant species which were well adapted to their environment. Generally, the rare species stayed rare under certain environments, however, there was a great possibility that the rare species become abundant when the environment become appropriate. For example, a rare marine microbe (i.e. *Prymnesium parvum* Carter) species could become abundant when aquatic environments changed to more conducive for its growth [31]. In this way, large numbers of rare species showed a stochastic distribution under complicated aquatic environments, but local environmental factors exerted a stronger influence on the globally important microeukaryotic communities.

### Comparison of community composition revealed by DGGE and Miseq sequencing

Past studies on microeukaryotic plankton community around Xiamen offshore area revealed the biomass, distribution and composition characteristics of prominent species mainly using microscopy [32], HPLC (high performance liquid chromatography) [33] and PCR**-**DGGE [11]. However, these studies have been mainly restricted to specific zooplankton or phytoplankton separately. Besides, only professional taxonomists can make morphological research reliable, and HPLC can merely investigate phytoplankton community [33–34]. Bao et al [11] only concentrated on eukaryotic ultraplankton assemblages by DGGE in western sea area of Xiamen Island in 2007. For identifying a larger range of species around Xiamen offshore area, we used both DGGE and Illumina Miseq sequencing methods to explore the characteristic of microeukaryotic plankton communities. Five important groups including Copepoda, Ciliophora, Chlorophyta, Dinophyceae, and Cryptophyta were detected at high taxonomical level with both methods, even though their relative abundance showed some small differences between techniques.

Our study offered novel insights into the community composition and genetic diversity of the microeukaryotic plankton in Xiamen offshore waters. However, it should be noticed that there are still some limitations in the DGGE technique. What usually occurs is that species with a low abundance may not clearly appear in distinct bands [35], thereby this approach misses a large number of rare species. It is usually believed that a species abundance of more than 1% of total abundance can be reliably detected in DGGE profiles [36]. Besides, not all the bands in DGGE pattern can be retrieved because sometimes they are not conspicuous enough to be recognized. Therefore DGGE can generate less information than Illumina Miseq sequencing [37]. In addition, Hong et al [38] proved that bands in the exactly same horizontal position on a DGGE gel may be not represent the same microorganism. Another study further proved this finding by confirming a band position covering several sequences representing different species [39]. In our study, we found that the bands B28, B31, B32, and B35 contained two different species or OTUs, respectively (S1 Table).

Over the past few years, high-throughput sequencing techniques have been substantially improved in quality and cost, therefore the next**-**generation sequencing (NGS) is becoming more widely used to examine the composition and dynamics of microeukaryotic communities in different ecosystems [5]. In our study, the microeukaryotic species richness revealed by Miseq sequencing was much greater than that established by PCR**-**DGGE (8,983 OTUs vs 73 bands). Interestingly, besides the five common phyla or dominant groups detected by two methods, we also found diatoms, chytridiomycota and some metazoan such as Annelida, Mollusca, Platyhelminthes and Rotifera by Miseq sequencing. Notably, diatoms were abundant in twelve sampling sties, this was consistent with past study that diatom was a dominant taxon in Xiamen sea water [33, 40]. In the same way, Bao et al [11] also did not detect diatom in Xiamen offshore area by PCR**-**DGGE method. The underrepresentation of diatom by PCR**-**DGGE can be due to the potential biases of PCR amplification and specific primers as well as clone steps [41].

Determining biodiversity in species-rich environments raises questions of whether the sampling effort was sufficient to fully capture the extent of the natural community. We obtained 8,983 OTUs by using Illumina Miseq sequencing which was unexpectedly higher than that achieved by DGGE, however, the total OTU rarefaction curve was far from saturated (S2 Fig). It might fail to find some species, especially the rare species, underestimating the real diversity of the microeukaryotic communities. However, most dominant species successfully retrieved by Miseq sequencing were consistent with the DGGE results and past studies, which would not markedly affect the pattern analysis of the whole microeukaryotic community.

The microeukaryotic communitie revealed by Miseq sequencing are nearly two orders of magnitude more diverse than those detected by DGGE. However, it should be noted that NGS errors may skew our view of the true complexity of microbial communities [42], which usually resulted in overestimates of taxon richness [43]. The much shorter reads generated by Illumina Miseq sequencing tend to contribute the richness overestimates. Behnke et al [44] indicated that the shorter reads in V9 region showed higher number of OTUs. However, for sequencing data sets of V9 region, OTU called at an appropriate sequencing similarity (95%) would offer the closest comparison with true OTU richness of protistan community. In our study, the relatively shorter reads and OTUs clustered at 97% similarity might show a slightly higher number than true OTU number. Further, the short sequences generated by Miseq sequencing were likely to be problematic for inferring phylogeny due to the small number of characters [5]. However, past study has proved that such short sequences are sufficient for accurate estimation of true communities [45].

### The protist dominance in north sites N2 and N3

Our Illumina Miseq sequencing results showed that some of the dominant species in sites N2 and N3 were obviously different from those of other sites (Fig 2B and S3 Fig), although more than 10,000 sequences were unclassified taxa at both sites and may represent previously unsampled diversity (S3 Table). Both sites N2 and N3 had fewer diatom species than other sites. It has been reported that most planktonic diatoms can survive between 5°C and 40°C, however the optimum temperature fluctuates between 15°C and 30°C, for instance, *Skeletonema costatum* (Grev.) Cleve, *Biddulphia sinensis* Greville, and *Streptotheca thamesis* Shrubsole can live better at 20**–**25°C, 25**–**28°C and 20**–**25°C, respectively [46]. The surface water temperature was high in twelve sites, particularly N2 and N3 sites measured in this study, here the surface water temperature reached a maximum value of over 31°C. The high temperature could promote the thermal stratification of water column in Xiamen offshore, thereby leading to the short supply of silicon nutrient. These results suggested that high temperature can decrease the diatom abundance in Xiamen offshore water in summer.

Interestingly, we found that five ciliate taxa (*Rimostrombidium*, *Halteria*, *Tintinnidium*, *Cryptocaryon* and *Pseudotontonia*), two cryptophytes (*Teleaulax* and *Cryptomonas*), one dinoflagellate (*Ceratium*) and one stramenopile (*Bicosoeca*) were abundant in sites N2 and N3. However, these taxa were rarely detected, or not dominant, in other sites in this investigation. It is worth noting that despite the 18S rDNA sequence being regularly used for studies of microeukaryote isolates, there are still some limitations. The major problem of employing the 18S rRNA genes as an amplifying target is the high copy number which usually becomes 10 to 100 times of a single**-**copy gene [47]. The high copy number usually influences accurate estimates of species relative abundance. For example, Behnke et al [48] found that alveolates such as ciliates were often dominant in molecular surveys however the ciliates often showed much lower percentages in morphological (microscope based) studies [41, 44, 49]. Thus, the ciliates overestimate might be interpreted by the SSU rRNA genes high copy number [50 **–** 51]. In addition, high copy number of SSU rRNA gene in dinoflagellate [52] and stramenopile [53] has been reported. However, this phenomenon has not been reported for cryptophytes. Using group**-**specific PCR primers and restricting the analysis to taxa with similar rDNA copy numbers may well solve this problem. However, Medinger et al [54] strongly advocated that the best strategy for future NGS projects is to develop single copy markers that could replace the rDNA sequences for protist community researches.

## Conclusions

Microeukaryotic plankton community composition showed great spatial variation in Xiamen offshore waters, especially at sampling sites N2 and N3, although their richness and diversity were not significantly different among the northern, eastern, southern and western stations based on both PCR**-**DGGE and Illumina MiSeq sequencing approaches. The Illumina MiSeq sequencing revealed almost two orders of magnitude higher taxon richness than did DGGE, as the former method can capture the vast low**-**abundance taxa. Redundancy analysis (RDA) showed that both temperature and salinity were the significant environmental factors influencing the distribution of dominant species (DGGE**-**based) communities, however the all (Illumina-based) microeukaryotic community might be affected by a complex of environmental factors which need further ecological study. Our study represents one of the first and most comprehensive examinations of marine microeukaryotic diversity in subtropical coastal water using the high-throughput sequencing technology. Our data will provide basic information for further studies on microeukaryotic community structure in waters around Xiamen Island and contribute to the sustainable management and conservation of local coastal ecosystems.

## Supporting Information

S1 Fig DGGE profile of 18S rRNA gene of microeukaryotic community from the Xiamen offshore sea area. (TIF)

S2 Fig Rarefaction curves of similarity-based operational taxonomic unit (OTUs). All these rarefaction analyses were based on Miseq sequencing of V9 hypervariable regions of the SSU rRNA gene at cluster distance value of 0.03. While the OTU number estimate for all samples is very similar, the combined data set resulted in substantially higher estimated of OTU number.
(TIF)

S3 Fig Cluster and heatmap of protist relative abundance for the twelve sampling sites. This is revealed by Miseq sequencing of V9 hypervariable region of the SSU rRNA gene.
(TIF)

S1 Table Phylogenetic affiliations of microeukaryotic plankton revealed by DGGE bands. (DOC)

S2 Table Top five most abundant OTUs in twelve sampling sites revealed by Miseq sequencing. (DOC)

S3 Table Phylum, OTU, sequence number of metazoa, protist, fungi and unclassified species revealed by Miseq sequencing. (DOC)
