# Supplementary figures and images for: Determining Microeukaryotic Plankton Community around Xiamen Island, Southeast China, Using Illumina MiSeq and PCR-DGGE Techniques
Source: PLoS One. 2015 May 28;10(5):e0127721. doi: 10.1371/journal.pone.0127721 (PMC4447373; doi:10.1371/journal.pone.0127721)

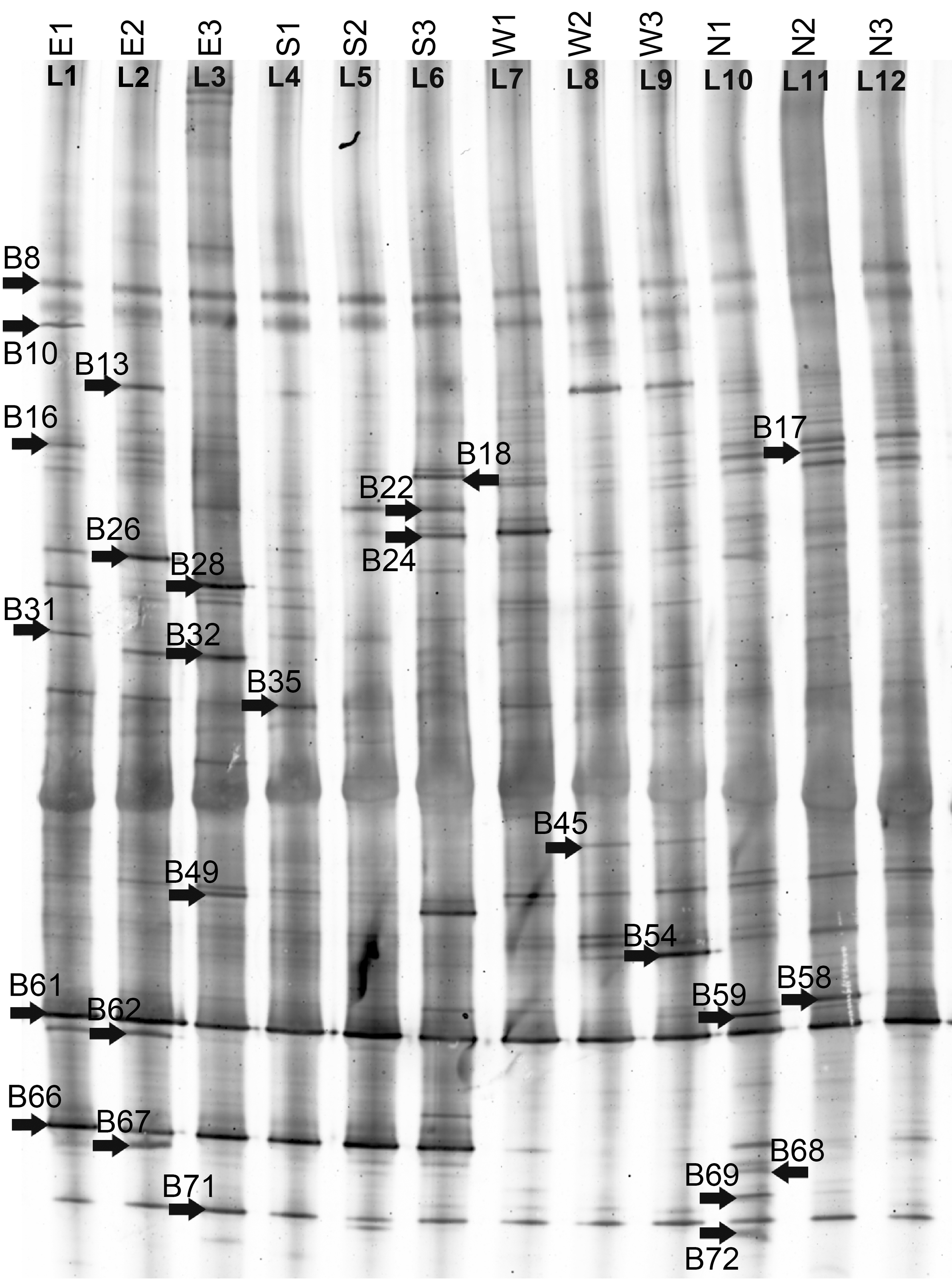

Supplement: S1 Fig — (TIF) [file pone.0127721.s001.tif]

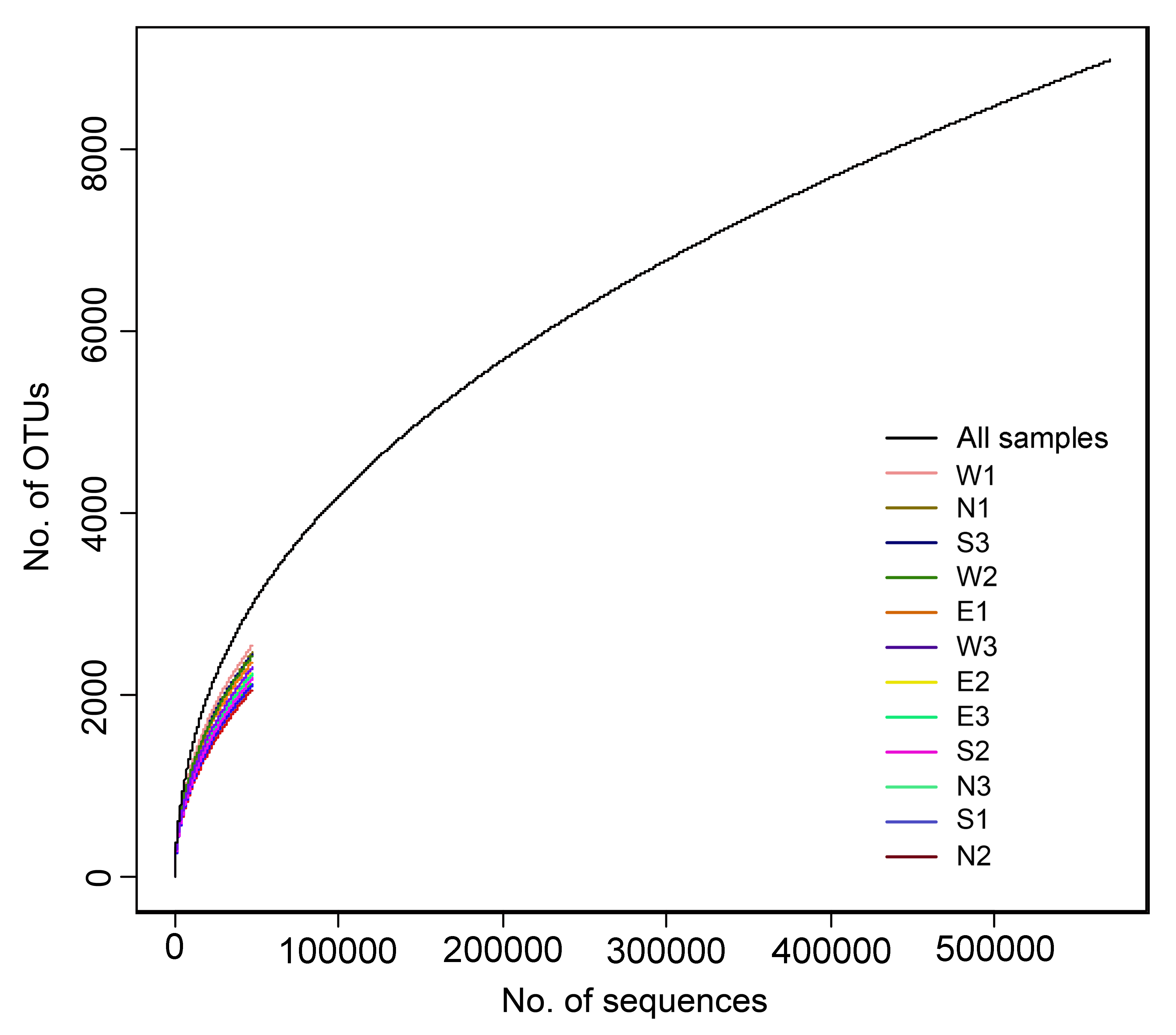

Supplement: S2 Fig — All these rarefaction analyses were based on Miseq sequencing of V9 hypervariable regions of the SSU rRNA gene at cluster distance value of 0.03. While the OTU number estimate for all samples is very similar, the combined data set resulted in substantially higher estimated of OTU number. (TIF) [file pone.0127721.s002.tif]

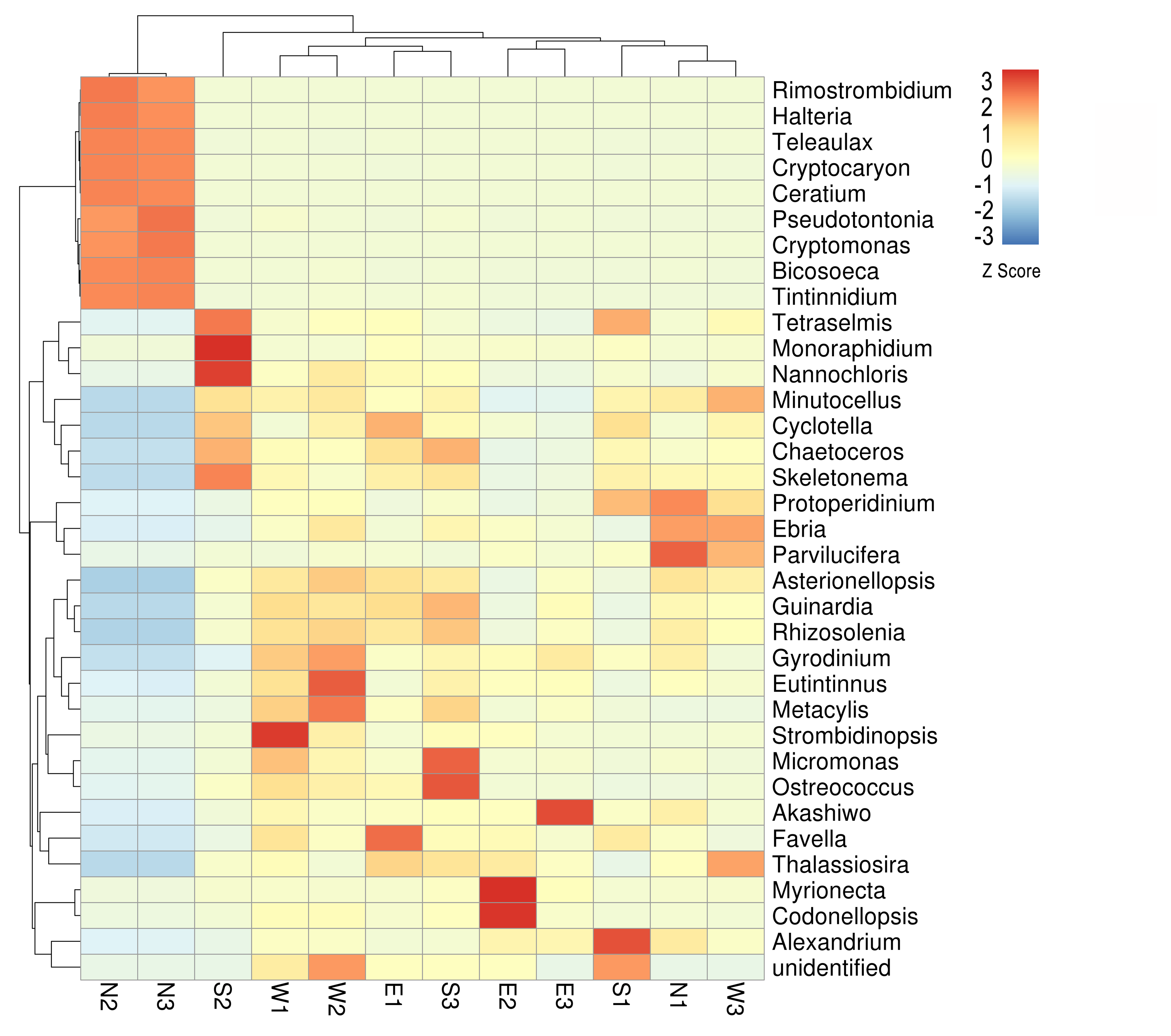

Supplement: S3 Fig — This is revealed by Miseq sequencing of V9 hypervariable region of the SSU rRNA gene. (TIF) [file pone.0127721.s003.tif]
