# Supplementary material for: Determining Microeukaryotic Plankton Community around Xiamen Island, Southeast China, Using Illumina MiSeq and PCR-DGGE Techniques
Source: PLoS One. 2015 May 28;10(5):e0127721. doi: 10.1371/journal.pone.0127721 (PMC4447373; doi:10.1371/journal.pone.0127721)
Supplement: S1 Table — (DOC) [file pone.0127721.s004.doc]

**Table S1.** Phylogenetic affiliations of microeukaryotic plankton revealed by DGGE bands.

| DGGE band | Accession number | Closest relative (accession number) | Similarity (%) | Source | Taxon |
| --- | --- | --- | --- | --- | --- |
| L1-B8-1 | KJ638859 | Uncultured eukaryote clone HA/BU4 18S rRNA gene (KC904751) | 99 | Marine | Copepoda, OTU1 |
| L1-B8-2 | KR019689 | Uncultured eukaryote clone DMS7 18S rRNA gene (JQ013187) | 99 | Marine | Copepoda, OTU1 |
| L1-B8-3 | KR019690 | Uncultured eukaryote clone HA/BU4 18S rRNA gene (KC904751) | 99 | Marine | Copepoda, OTU1 |
| L1-B10-1 | KJ638860 | Uncultured eukaryote clone JLRE-15-2 18S rRNA gene (JF260910) | 99 | Marine | Copepoda, OTU2 |
| L1-B10-2 | KR019691 | Uncultured eukaryote clone JLRE-15-2 18S rRNA gene (JF260910) | 99 | Marine | Copepoda, OTU2 |
| L1-B10-3 | KR019692 | Uncultured eukaryote clone JLRE-15-2 18S rRNA gene (JF260910) | 99 | Marine | Copepoda, OTU2 |
| L4-B35-1 | KJ638861 | Uncultured eukaryote clone JLRE-15-2 18S rRNA gene (JF260910) | 99 | Marine | Copepoda, OTU2 |
| L4-B35-2 | KR019699 | Uncultured eukaryote clone JLRE-15-2 18S rRNA gene (JF260910) | 99 | Marine | Copepoda, OTU2 |
| L1-B61-1 | KJ638862 | Uncultured eukaryote clone JLRE-15-2 18S rRNA gene (JF260910) | 99 | Marine | Copepoda, OTU2 |
| L1-B61-2 | KR019700 | Uncultured eukaryote clone JLRE-15-2 18S rRNA gene (JF260910) | 98 | Marine | Copepoda, OTU2 |
| L1-B61-3 | KR019701 | Uncultured eukaryote clone JLRE-15-2 18S rRNA gene (JF260910) | 98 | Marine | Copepoda, OTU2 |
| L2-B62-1 | KJ638863 | Uncultured eukaryote clone JLRE-15-2 18S rRNA gene (JF260910) | 98 | Marine | Copepoda, OTU3 |
| L2-B62-2 | KR019702 | Uncultured eukaryote clone JLRE-15-2 18S rRNA gene (JF260910) | 97 | Marine | Copepoda, OTU3 |
| L2-B62-3 | KR019703 | Uncultured eukaryote clone JLRE-15-2 18S rRNA gene (JF260910) | 96 | Marine | Copepoda, OTU3 |
| L1-B66-1 | KJ638864 | Uncultured eukaryote clone G1029.0260-D66 18S rRNA gene (KF918265) | 100 | Marine | Copepoda, OTU4 |
| L1-B66-2 | KR019704 | Uncultured eukaryote clone G1029.0202-D8 18S rRNA gene (KF918209) | 99 | Marine | Copepoda, OTU4 |
| L1-B66-3 | KR019705 | Uncultured eukaryote clone G1029.0202-D8 18S rRNA gene (KF918209) | 100 | Marine | Copepoda, OTU4 |
| L2-B67-1 | KJ638865 | Uncultured eukaryote clone G1029.0260-D66 18S rRNA gene (KF918265) | 100 | Marine | Copepoda, OTU4 |
| L2-B67-2 | KR019706 | Uncultured eukaryote clone G1029.0202-D8 18S rRNA gene (KF918209) | 99 | Marine | Copepoda, OTU4 |
| L2-B67-3 | KR019707 | Uncultured eukaryote clone G1029.0202-D8 18S rRNA gene (KF918209) | 99 | Marine | Copepoda, OTU4 |
| L3-B71-1 | KJ638866 | Uncultured eukaryote clone HA/BU4 18S rRNA gene (KC904751) | 99 | Marine | Copepoda, OTU1 |
| L3-B71-2 | KR019708 | Uncultured eukaryote clone DMS7 18S rRNA gene (JQ013187) | 99 | Marine | Copepoda, OTU1 |
| L3-B71-3 | KR019709 | Uncultured eukaryote clone DMS7 18S rRNA gene (JQ013187) | 99 | Marine | Copepoda, OTU1 |
| L10-B69-1 | KJ638867 | Uncultured eukaryote clone HA/BU4 18S rRNA gene (KC904751) | 99 | Marine | Copepoda, OTU1 |
| L10-B69-2 | KR019729 | Uncultured eukaryote clone HA/BU4 18S rRNA gene (KC904751) | 99 | Marine | Copepoda, OTU1 |
| L10-B69-3 | KR019730 | Uncultured eukaryote clone HA/BU4 18S rRNA gene (KC904751) | 99 | Marine | Copepoda, OTU1 |
| L10-B72-1 | KJ638868 | Uncultured eukaryote clone JLRE-15-2 18S rRNA gene (JF260910) | 99 | Marine | Copepoda, OTU2 |
| L10-B72-2 | KR019731 | Uncultured eukaryote clone JLRE-15-2 18S rRNA gene (JF260910) | 99 | Marine | Copepoda, OTU2 |
| L10-B72-3 | KR019732 | Uncultured eukaryote clone JLRE-15-2 18S rRNA gene (JF260910) | 99 | Marine | Copepoda, OTU2 |
| L1-B16-1 | KJ638869 | *Desmodesmus subspicatus* strain H 18S rRNA gene (HQ834485) | 99 | Marine | Chlorophyta, OTU5 |
| L1-B16-2 | KR019693 | Uncultured chlorophyte clone G24 18S rRNA gene (EU910612) | 99 | Marine | Chlorophyta, OTU5 |
| L1-B16-3 | KR019694 | Uncultured chlorophyte clone G24 18S rRNA gene (EU910612) | 99 | Marine | Chlorophyta, OTU5 |
| L2-B26-1 | KJ638870 | Uncultured marine eukaryote clone SGUH1199.FRAG.MO.5m 18S rRNA gene (JX841442) | 100 | Marine | Chlorophyta, OTU6 |
| L2-B26-2 | KR019695 | Uncultured marine eukaryote clone SGUH1199.FRAG.MO.5m 18S rRNA gene (JX841442) | 99 | Marine | Chlorophyta, OTU6 |
| L2-B26-3 | KR019696 | Uncultured marine eukaryote clone SGUH1199.FRAG.MO.5m 18S rRNA gene (JX841442) | 99 | Marine | Chlorophyta, OTU6 |
| L3-B28-1 | KJ638871 | *Chlorella sorokiniana* strain Icheon4 18S rRNA gene (KF864476) | 99 | Marine | Chlorophyta, OTU6 |
| L3-B32-1 | KJ638872 | Uncultured phytoplankton clone Q3-17 18S rRNA gene (JQ420114) | 98 | Marine | Chlorophyta, OTU7 |
| L3-B32-2 | KR019712 | Uncultured phytoplankton clone Q3-17 18S rRNA gene (JQ420114) | 98 | Marine | Chlorophyta, OTU7 |
| L11-B58-1 | KJ638873 | Uncultured marine eukaryote clone BTPL20040810.0020 18S rRNA gene (FJ222093) | 99 | Marine | Chlorophyta, OTU8 |
| L11-B58-2 | KR019725 | Uncultured Chlorophyta clone G0Esp_4_29 18S rRNA gene (KC911760) | 99 | Marine | Chlorophyta, OTU8 |
| L11-B58-3 | KR019726 | Uncultured Chlorophyta clone G0Esp_4_29 18S rRNA gene (KC911760) | 99 | Marine | Chlorophyta, OTU8 |
| L1-B31-1 | KJ638874 | Uncultured eukaryote clone JLRE-14-4 18S rRNA gene (JF260907) | 98 | Marine | Cryptophyta, OTU9 |
| L1-B31-2 | KR019698 | Uncultured eukaryote clone JLRE-14-4 18S rRNA gene (JF260907) | 98 | Marine | Cryptophyta, OTU9 |
| L10-B68-1 | KJ638875 | Uncultured eukaryote clone JLRE-14-4 18S rRNA gene (JF260907) | 98 | Marine | Cryptophyta, OTU10 |
| L10-B68-2 | KR019727 | Uncultured eukaryote clone JLRE-14-4 18S rRNA gene (JF260907) | 98 | Marine | Cryptophyta, OTU10 |
| L10-B68-3 | KR019728 | Uncultured eukaryote clone JLRE-14-4 18S rRNA gene (JF260907) | 98 | Marine | Cryptophyta, OTU10 |
| L2-B13-1 | KJ638876 | Uncultured eukaryote clone ES_127 18S rRNA gene (JN979514) | 99 | Marine | Ciliophora, OTU11 |
| L2-B13-2 | KR019710 | Uncultured marine eukaryote clone SGUH558.FRAG.MO.5m 18S rRNA gene (JX841724) | 98 | Marine | Ciliophora, OTU11 |
| L2-B13-3 | KR019711 | Uncultured eukaryote clone SGUH558 18S rRNA gene (KJ763175) | 98 | Marine | Ciliophora, OTU11 |
| L3-B28-2 | KJ638877 | Uncultured marine eukaryote clone DP-084 18S rRNA gene (KC539448) | 99 | Marine | Ciliophora, OTU12 |
| L3-B28-3 | KR019697 | Uncultured marine eukaryote clone DP-084 18S rRNA gene (KC539448) | 98 | Marine | Ciliophora, OTU12 |
| L3-B32-3 | KJ638878 | Uncultured marine eukaryote clone DP-084 18S rRNA gene (KC539448) | 99 | Marine | Ciliophora, OTU12 |
| L6-B22-1 | KJ638879 | Uncultured marine eukaryote clone SGUH558.FRAG.MO.5m 18S rRNA gene (JX841724) | 98 | Marine | Ciliophora, OTU13 |
| L6-B22-2 | KR019717 | Uncultured eukaryote clone SGUH558 18S rRNA gene (KJ763175) | 98 | Marine | Ciliophora, OTU13 |
| L6-B22-3 | KR019718 | Uncultured marine eukaryote clone SGUH1297.FRAG.MO.5m 18S rRNA gene (JX841489) | 98 | Marine | Ciliophora, OTU13 |
| L6-B24-1 | KJ638880 | *Favella ehrenbergii* isolate c07 18S rRNA gene (GU574770) | 99 | Marine | Ciliophora, OTU14 |
| L6-B24-2 | KR019719 | *Favella ehrenbergii* isolate c13 18S rRNA gene (GU574769) | 99 | Marine | Ciliophora, OTU14 |
| L6-B24-3 | KR019720 | *Favella panamensis* 18S rRNA gene (AY143572) | 99 | Marine | Ciliophora, OTU14 |
| L3-B49-1 | KJ638881 | Uncultured eukaryote clone G1029.0153-C26 18S rRNA gene (KF918164) | 99 | Marine | Dinophyceae, OTU15 |
| L3-B49-2 | KR019713 | Uncultured eukaryote clone G1029.0153-C26 18S rRNA gene (KF918164) | 99 | Marine | Dinophyceae, OTU15 |
| L3-B49-3 | KR019714 | Uncultured eukaryote clone SGYH416 18S rRNA gene (KJ762630) | 99 | Marine | Dinophyceae, OTU15 |
| L6-B18-1 | KJ638882 | Uncultured marine eukaryote clone SGYH855.FRAG.MO.500m 18S rRNA gene (JX842602) | 99 | Marine | Dinophyceae, OTU15 |
| L6-B18-2 | KR019715 | Uncultured eukaryote clone G1029.0153-C26 18S rRNA gene (KF918164) | 99 | Marine | Dinophyceae, OTU15 |
| L6-B18-3 | KR019716 | Uncultured eukaryote clone G1029.0153-C26 18S rRNA gene (KF918164) | 99 | Marine | Dinophyceae, OTU15 |
| L9-B54-1 | KJ638883 | *Protoperidinium excentricum* small subunit rRNA gene (AY443021) | 99 | Marine | Dinophyceae, OTU16 |
| L9-B54-2 | KR019721 | *Protoperidinium excentricum* small subunit rRNA gene (AY443021) | 99 | Marine | Dinophyceae, OTU16 |
| L9-B54-3 | KR019722 | *Protoperidinium excentricum* small subunit rRNA gene (AY443021) | 98 | Marine | Dinophyceae, OTU16 |
| L10-B59-1 | KJ638884 | *Protoperidinium excentricum* small subunit rRNA gene (AY443021) | 91 | Marine | Dinophyceae, OTU17 |
| L10-B59-2 | KR019723 | *Protoperidinium excentricum* small subunit rRNA gene (AY443021) | 91 | Marine | Dinophyceae, OTU17 |
| L10-B59-3 | KR019724 | *Protoperidinium excentricum* small subunit rRNA gene (AY443021) | 91 | Marine | Dinophyceae, OTU17 |
| L4-B35-3 | KJ638885 | *Alexandrium* sp. tamutum 18S rRNA gene, strain AT5 (AJ535377) | 99 | Marine | Dinophyceae, OTU18 |
| L1-B31-3 | KJ638886 | *Akashiwo sanguinea* isolate GSW0207 small subunit rRNA gene (AY421770) | 99 | Marine | Dinophyceae, OTU19 |
| L8-B45-1 | KJ638887 | Uncultured eukaryote clone F3-056 18S rRNA gene (KF378097) | 95 | Marine | Unknown eukaryote, OTU20 |
| L8-B45-2 | KR019733 | Uncultured eukaryote clone F3-025 18S rRNA gene (KF378096) | 95 | Marine | Unknown eukaryote, OTU20 |
| L8-B45-3 | KR019734 | Uncultured eukaryote clone F3-025 18S rRNA gene (KF378096) | 95 | Marine | Unknown eukaryote, OTU20 |
| L11-B17-1 | KJ638888 | Uncultured eukaryote isolate DGGE gel band JLJ-8-12 18S rRNA gene (JN846849) | 95 | Marine | Rhizaria, OTU21 |
| L11-B17-2 | KR019735 | Uncultured eukaryote isolate DGGE gel band JLJ-8-12 18S rRNA gene (JN846849) | 95 | Marine | Rhizaria, OTU21 |
| L11-B17-3 | KR019736 | Uncultured eukaryote isolate DGGE gel band JLJ-8-12 18S rRNA gene (JN846849) | 95 | Marine | Rhizaria, OTU21 |
