# Supplementary material for: Determining Microeukaryotic Plankton Community around Xiamen Island, Southeast China, Using Illumina MiSeq and PCR-DGGE Techniques
Source: PLoS One. 2015 May 28;10(5):e0127721. doi: 10.1371/journal.pone.0127721 (PMC4447373; doi:10.1371/journal.pone.0127721)
Supplement: S2 Table — (DOC) [file pone.0127721.s005.doc]

**Table S2.** Top five most abundant OTUs in twelve sampling sites revealed by Miseq sequencing.

| Site | OTU ID | Percentage  (%) | Taxon | Similarity  (%) | Closest relative (accession number) |
| --- | --- | --- | --- | --- | --- |
| N1 | 1.OTU_1 | 10.05% | Arthropoda/Copepoda | 94% | *Acartia longiremis* (GU969156) |
|  | 2.OTU_2 | 7.05% | Arthropoda/Copepoda | 93% | *Acartia longiremis* (GU969156) |
|  | 3.OTU_10973 | 4.26% | Bacillariophyta | 99% | *Thalassiosira lundiana* (HM991692) |
|  | 4.OTU_7 | 4.21% | Mammalia/Craniata | 99% | *Oryctolagus cuniculus* (NR033238) |
|  | 5.OTU_3 | 3.80% | Bacillariophyta | 99% | *Thalassiosira eccentrica* (JQ217343) |
| N2 | 1.OTU_9 | 5.78% | Arthropoda/Copepoda | 97% | *Skistodiaptomus pygmaeus* (AY339161) |
|  | 2.OTU_10 | 5.69% | Arthropoda/Copepoda | 97% | *Skistodiaptomus pallidus* (AY339160) |
|  | 3.OTU_13 | 4.82% | Apicomplexa/Perkinsidae | 92% | *Perkinsidae* sp. (JX869394) |
|  | 4.OTU_14 | 4.72% | Apicomplexa/Perkinsidae | 92% | *Perkinsidae* sp. (JX869393) |
|  | 5.OTU_17 | 4.01% | Mastigophora | 91% | *Sphaeroeca volvox* (Z34900) |
| N3 | 1.OTU_10 | 5.87% | Arthropoda/Copepoda | 97% | *Skistodiaptomus pallidus* (AY339160) |
|  | 2.OTU_9 | 5.67% | Arthropoda/Copepoda | 97% | *Skistodiaptomus pygmaeus* (AY339161) |
|  | 3.OTU_13 | 4.88% | Apicomplexa/Perkinsidae | 92% | *Perkinsidae* sp. (JX869394) |
|  | 4.OTU_14 | 4.27% | Apicomplexa/Perkinsidae | 92% | *Perkinsidae* sp. (JX869393) |
|  | 5.OTU_17 | 4.16% | Mastigophora | 91% | *Sphaeroeca volvox* (Z34900) |
| E1 | 1.OTU_10973 | 7.78% | Bacillariophyta | 99% | *Thalassiosira lundiana* (HM991692) |
|  | 2.OTU_3 | 6.94% | Bacillariophyta | 99% | *Thalassiosira eccentrica* (JQ217343) |
|  | 3.OTU_1 | 5.58% | Arthropoda/Copepoda | 94% | *Acartia longiremis* (GU969156) |
|  | 4.OTU_2 | 5.43% | Arthropoda/Copepoda | 93% | *Acartia longiremis* (GU969156) |
|  | 5.OTU_7 | 2.84% | Mammalia/Craniata | 99% | *Oryctolagus cuniculus* (NR033238) |
| E2 | 1.OTU_5 | 10.06% | Arthropoda/Copepoda | 99% | *Oithona davisae* (KJ814022) |
|  | 2.OTU_6 | 8.02% | Arthropoda/Copepoda | 92% | *Oithona* sp. (JF781539) |
|  | 3.OTU_10973 | 6.75% | Bacillariophyta | 99% | *Thalassiosira lundiana* (HM991692) |
|  | 4.OTU_1 | 6.51% | Arthropoda/Copepoda | 94% | *Acartia longiremis* (GU969156) |
|  | 5.OTU_3 | 5.70% | Bacillariophyta | 99% | *Thalassiosira eccentrica* (JQ217343) |
| E3 | 1.OTU_1 | 17.36% | Arthropoda/Copepoda | 94% | *Acartia longiremis* (GU969156) |
|  | 2.OTU_2 | 13.78% | Arthropoda/Copepoda | 93% | *Acartia longiremis* (GU969156) |
|  | 3.OTU_5 | 6.15% | Arthropoda/Copepoda | 99% | *Oithona davisae* (KJ814022) |
|  | 4.OTU_6 | 4.84% | Arthropoda/Copepoda | 92% | *Oithona* sp. (JF781539) |
|  | 5.OTU_10973 | 4.16% | Bacillariophyta | 99% | *Thalassiosira lundiana* (HM991692) |
| S1 | 1.OTU_1 | 14.59% | Arthropoda/Copepoda | 94% | *Acartia longiremis* (GU969156) |
|  | 2.OTU_2 | 9.79% | Arthropoda/Copepoda | 93% | *Acartia longiremis* (GU969156) |
|  | 3.OTU_5 | 7.44% | Arthropoda/Copepoda | 99% | *Oithona davisae* (KJ814022) |
|  | 4.OTU_6 | 4.65% | Arthropoda/Copepoda | 92% | *Oithona* sp. (JF781539) |
|  | 5.OTU_12 | 2.31% | Bacillariophyta | 100% | *Cyclotella choctawhatcheeana* (JF791071) |
| S2 | 1.OTU_25 | 6.39% | Chlorophyta | 99% | *Chlorellaceae* sp. (FM205844) |
|  | 2.OTU_26 | 6.34% | Chlorophyta | 95% | *Dictyosphaerium* sp*.* (GQ487254) |
|  | 3.OTU_3 | 3.48% | Bacillariophyta | 99% | *Thalassiosira eccentrica* (JQ217343) |
|  | 4.OTU_5 | 3.41% | Arthropoda/Copepoda | 99% | *Oithona davisae* (KJ814022) |
|  | 5.OTU_2 | 3.31% | Arthropoda/Copepoda | 93% | *Acartia longiremis* (GU969156) |
| S3 | 1.OTU_1 | 7.17% | Arthropoda/Copepoda | 94% | *Acartia longiremis*(GU969156) |
|  | 2.OTU_2 | 7.04% | Arthropoda/Copepoda | 93% | *Acartia longiremis* (GU969156) |
|  | 3.OTU_10973 | 6.81% | Bacillariophyta | 99% | *Thalassiosira lundiana* (HM991692) |
|  | 4.OTU_3 | 5.79% | Bacillariophyta | 99% | *Thalassiosira eccentrica* (JQ217343) |
|  | 5.OTU_7 | 2.14% | Mammalia/Craniata | 99% | *Oryctolagus cuniculus* (NR033238) |
| W1 | 1.OTU_1 | 10.82% | Arthropoda/Copepoda | 94% | *Acartia longiremis* (GU969156) |
|  | 2.OTU_2 | 8.18% | Arthropoda/Copepoda | 93% | *Acartia longiremis* (GU969156) |
|  | 3.OTU_10973 | 4.25% | Bacillariophyta | 99% | *Thalassiosira lundiana* (HM991692) |
|  | 4.OTU_3 | 4.15% | Bacillariophyta | 99% | *Thalassiosira eccentrica* (JQ217343) |
|  | 5.OTU_7 | 2.35% | Mammalia/Craniata | 99% | *Oryctolagus cuniculus* (NR033238) |
| W2 | 1.OTU_1 | 9.44% | Arthropoda/Copepoda | 94% | *Acartia longiremis* (GU969156) |
|  | 2.OTU_2 | 6.77% | Arthropoda/Copepoda | 93% | *Acartia longiremis* (GU969156) |
|  | 3.OTU_16 | 4.68% | Arthropoda/Copepoda | 94% | *Calanus helgolandicus* (JX995318) |
|  | 4.OTU_11 | 3.34% | Arthropoda/Copepoda | 99% | *Acartia longiremis* (GU969156) |
|  | 5.OTU_10973 | 3.22% | Bacillariophyta | 99% | *Thalassiosira lundiana* (HM991692) |
| W3 | 1.OTU_10973 | 10.40% | Bacillariophyta | 99% | *Thalassiosira lundiana* (HM991692) |
|  | 2.OTU_3 | 8.03% | Bacillariophyta | 99% | *Thalassiosira eccentrica* (JQ217343) |
|  | 3.OTU_1 | 5.34% | Arthropoda/Copepoda | 94% | *Acartia longiremis* (GU969156) |
|  | 4.OTU_2 | 5.15% | Arthropoda/Copepoda | 93% | *Acartia longiremis* (GU969156) |
|  | 5.OTU_7 | 4.11% | Mammalia/Craniata | 99% | *Oryctolagus cuniculus* (NR033238) |
