# Supplementary material for: Determining Microeukaryotic Plankton Community around Xiamen Island, Southeast China, Using Illumina MiSeq and PCR-DGGE Techniques
Source: PLoS One. 2015 May 28;10(5):e0127721. doi: 10.1371/journal.pone.0127721 (PMC4447373; doi:10.1371/journal.pone.0127721)
Supplement: S3 Table — (DOC) [file pone.0127721.s006.doc]

**Table S3.** Phylum, OTU, sequence number of metazoa, protist, fungi and unclassified species revealed by Miseq sequencing.

| Taxon |  | N1 | N2 | N3 | E1 | E2 | E3 | S1 | S2 | S3 | W1 | W2 | W3 |
| --- | --- | --- | --- | --- | --- | --- | --- | --- | --- | --- | --- | --- | --- |
| Metazoa | phylum | 9 | 7 | 7 | 10 | 9 | 9 | 10 | 7 | 12 | 10 | 11 | 9 |
|  | OTU | 380 | 243 | 235 | 329 | 371 | 396 | 353 | 288 | 333 | 350 | 382 | 333 |
|  | sequence | 19086 | 12991 | 11545 | 13551 | 21713 | 26880 | 23855 | 10425 | 14576 | 16561 | 20050 | 13513 |
| Protist | phylum | 16 | 22 | 20 | 15 | 17 | 15 | 16 | 16 | 18 | 18 | 18 | 20 |
|  | OTU | 1462 | 1143 | 1151 | 1461 | 1425 | 1338 | 1180 | 1314 | 1665 | 1513 | 1482 | 1385 |
|  | sequence | 23584 | 23158 | 23065 | 29392 | 23124 | 17504 | 18899 | 31072 | 30896 | 25138 | 23798 | 29620 |
| Fungi | phylum | 3 | 3 | 3 | 3 | 3 | 3 | 3 | 3 | 3 | 3 | 3 | 4 |
|  | OTU | 22 | 71 | 74 | 16 | 16 | 19 | 18 | 13 | 17 | 21 | 21 | 26 |
|  | sequence | 627 | 661 | 666 | 120 | 109 | 110 | 104 | 94 | 189 | 197 | 326 | 493 |
| Unclassified | OTU | 612 | 609 | 673 | 564 | 484 | 490 | 566 | 575 | 433 | 670 | 554 | 564 |
|  | sequence | 4196 | 10683 | 12217 | 4430 | 2547 | 2999 | 4635 | 5902 | 1832 | 5597 | 3319 | 3867 |
